# An emergency-department-initiated outreach program for patients with opioid use disorder is associated with an increase in agonist therapy and engagement in addictions care: a one-year cohort study

**DOI:** 10.1186/s13011-023-00578-3

**Published:** 2024-02-21

**Authors:** Rukaiyah Lakkadghatwala, Daniel Lane, Frank Scheuermeyer, Jesse Hilburt, Jane Buxton, Cheyenne Johnson, Seonaid Nolan, Christy Sutherland, Jessica Moe, Raoul Daoust, Kathryn Dong, Jim Christenson, Isabelle Miles, Aaron Orkin, Madelyn Whyte, Andrew Kestler

**Affiliations:** 1 Department of Emergency Medicine, Surrey Memorial Hospital & Richmond Hospital, University of British Columbia, Vancouver, Canada; 2 Department of Emergency Medicine, University of British Columbia, Vancouver, Canada; 3 Department of Emergency Medicine & St. Paul’s Hospital, University of British Columbia, Vancouver, Canada; 4 Vancouver Coastal Health Overdose Outreach Team & St. Paul’s Hospital, Vancouver, Canada; 5 School of Population and Public Health & BC Centre for Disease Control, University of British Columbia, Vancouver, Canada; 6 BC Centre on Substance Use & University of British Columbia School of Nursing, Vancouver, Canada; 7 Department of Medicine & BC Centre on Substance Use, University of British Columbia, Vancouver, Canada; 8 Department of Family Practice, PHS Community Services Society & BC Centre on Substance Use, University of British Columbia, Vancouver, Canada; 9 Department of Emergency Medicine, BC Centre for Disease Control & Vancouver General Hospital, University of British Columbia, Vancouver, Canada; 10 Département Médecine de Famille Et Médecine d’Urgence, Université de Montréal, Hôpital Sacré-Coeur de Montréal & CIUSSS Nord-de-L’ile, Montreal, Canada; 11 Department of Emergency Medicine, University of Alberta, Edmonton, Canada; 12 Department of Family & Community Medicine, Inner City Health Associates Toronto & St. Joseph’s Health Centre, University of Toronto, Toronto, Canada; 13 Foundry BC, Vancouver, Canada; 14 Department of Emergency Medicine, Vancouver Coastal Health, BC Centre on Substance Use & St. Paul’s Hospital, University of British Columbia, Vancouver, Canada

**Keywords:** Addiction, Opioid, Opioid use disorder, Emergency medicine, Opioid agonist therapy, Prospective cohort study, Substance use disorder

## Abstract

**Background:**

People with opioid use disorder (OUD) are high-risk for short-term mortality and morbidity. Emergency department (ED) interventions can reduce those risks, but benefits wane without ongoing community follow-up.

**Objective:**

To evaluate an ED-based intensive community outreach program.

**Methods:**

At two urban EDs between October 2019 and March 2020, we enrolled patients with OUD not currently on opioid agonist therapy (OAT) in a prospective cohort study evaluating a one-year intensive community outreach program, which provided ongoing addictions care, housing resources, and community support. We surveyed patients at intake and at scheduled outreach encounters at one, two, six, and twelve months. Follow-up surveys assessed OAT uptake, addictions care engagement, housing status, quality of life scores, illicit opioid use, and outreach helpfulness. We used descriptive statistics for each period and conducted sensitivity and subgroup analyses to account for missing data.

**Results:**

Of 84 baseline participants, 29% were female and 32% were housed, with a median age of 33. Sixty participants (71%) completed at least one follow-up survey. Survey completion rates were 37%, 38%, 39%, and 40% respectively at one, two, six, and twelve months. Participants had a median of three outreach encounters. Among respondents, OAT was 0% at enrolment and ranged from 38% to 56% at follow-up; addictions care engagement was 22% at enrolment and ranged from 65% to 81% during follow-up; and housing was 40% at enrolment and ranged from 48% to 59% during follow-up. Improvements from baseline to follow-up occurred for all time periods. OAT and engagement in care benefits were maintained in sensitivity and subgroup analyses. Respondents rated the outreach program as helpful at all time periods,

**Conclusion:**

An ED-initiated intensive outreach program for patients with OUD not yet on OAT was associated with a persistent increase in OAT use and engagement in care, as well as housing.

**Supplementary Information:**

The online version contains supplementary material available at 10.1186/s13011-023-00578-3.

## Introduction

Mortality rates due to opioid use continue to rise in Canada, with the highest rates reported in British Columbia (BC) [1]. Opioid use also increases morbidity, as reflected in increasing opioid-related hospitalizations and emergency department (ED) visits [2]. EDs are a main access point of care for people who use opioids [2, 3]. In BC, 60% of patients experiencing an illicit drug overdose event have visited an ED in the prior year, compared to 17% of a matched control group not experiencing an overdose [3].

Opioid agonist therapy (OAT) reduces the morbidity and mortality risks related to opioid use [4, 5]. While initiating OAT in the ED increases retention in care compared to referral to addictions services alone, long term retention on OAT and in care is poor [6]. Ongoing community follow-up after OAT initiation in the ED, including OAT primary care visits, improves retention [7, 8]. The benefits of ED interventions wane without this ongoing community follow-up [7, 8]. Regardless of setting of presentation, OUD is a chronic relapsing disease with frequent OAT discontinuations [4, 5, 9].

In Vancouver, BC, ED patients who experience an overdose or who initiate OAT receive referral to a community outreach team [10]. This community outreach team attempts contacting the patient within a few days of the ED visit to provide linkages to community care and social supports with a 96% success rate contacting referred patients and a 98% success rate connecting patients to care and social services based on the initial referral [10]. However, the outreach team does not provide longitudinal follow-up. To improve follow up and with the hope of improving outcomes for ED patients with OUD, we designed an intensive ED-initiated community outreach program with four planned community outreach contacts over one year—at one, two, six, and twelve months after the ED visit. These contacts occurred in addition to the standard of care initial contact within a few days of the ED visit. We anticipated this more intensive approach—involving four additional outreach encounters over one year following the ED visit—would be associated with improved OAT uptake, engagement in care, and other health and social outcomes.

## Methods

### Setting

We conducted a prospective evaluation of an intensive outreach program initiated at two urban teaching EDs in Vancouver, British Columbia, each with over 85,000 annual patient visits. The University of British Columbia Ethics Board approved this study. Enrollment started October 2019 and ended early in March 2020 due to the COVID pandemic.

### Participants

We included ED patients aged at least 18 years old with confirmed OUD (see criteria below) not on OAT at the time of the ED visit (defined as not having taken OAT in the past 5 days).We excluded patients with moderate to severe opioid withdrawal requiring immediate treatment, residence outside of the health authority catchment area, pregnancy, medical contraindications to OAT, admission to hospital, police custody, and medical or psychiatric instability.

### Study procedures

Research assistants approached patients meeting screening criteria (previously on OAT, opioid related chief complaint, opioid use mentioned in triage note, or ED provider referral) and invited them to participate in the intensive outreach program. Patients declining participation were eligible for routine outreach referrals and all other ED-based OUD interventions. OUD was confirmed by Rapid Opioids Dependence Screen (RODS) criteria [11]. Participants provided informed consent to complete an intake survey and follow-up surveys during scheduled outreach at one, two, six, and twelve months (see Additional file 1 for intake and follow-up questionnaires). All patients were contacted within a few days of the ED visit for the standard of care single outreach encounter. Participants were then also contacted one, two, six, and twelve months after the ED visit for the four additional follow-up outreach encounters involved in this outreach program. The outreach team attempted at least three contact attempts by phone or in person based on the participant’s preference at each time point. Follow-up contact attempts included e-mail, text, phone calls, and in-person visits to residences, shelters, parks, safe injection sites, and any other place the participants were known to spend time. To locate patients, outreach staff also benefited from extensive community experience and had access to community care databases that contained information on prior outreach contacts, deaths, incarcerations, and residence changes to areas outside of the catchment area.

At each follow-up encounter, the outreach team offered services and community linkages (including addictions care, housing support, and additional needs outlined by the patient) in person or by telephone. At the same follow-up encounter, research staff embedded in the regional outreach team administered follow-up surveys. Participants could accept outreach services and decline survey participation at any given encounter. Research staff logged all outreach contacts, with or without survey completion.

Survey domains included demographics, drug use as measured by self-reported seven-day recall (timeline followback method) [12], engagement in care, current OAT status, treatment motivation score, health related quality of life as measured by Euroqol [13], housing status, and perceived helpfulness of the outreach program. Engagement in care was defined as a voluntary addictions care encounter (clinic, treatment centre, or detoxification centre) in the one week prior to the survey. Fixed housing was defined as residence in single-room occupancy hotel (SRO) or one’s own house or apartment. Two research team members with lived experience of OUD pilot tested the survey and edited it for clarity. Participants received $20 CAD in compensation for each survey completed.

Data analysis: Questionnaire responses were recorded on paper by research assistants, then entered into a REDCap database, and finally exported to Excel for analysis. We analyzed data with descriptive statistics, presenting medians (with interquartile ranges or IQR) or proportions for each time period. For comparison between periods, we calculated individual differences for scores, then the median of differences with IQR. For proportional comparisons between periods, we calculated the difference of proportions. We anticipated that not all participants would complete all surveys and that participants completing surveys at one time period might not be the same as those completing surveys at another time period. Comparing participants with complete data at each time point would result in comparing different groups of patients, potentially limiting the study’s ability to draw conclusions about group outcomes or individual outcomes over time. Therefore, in addition to comparing overall outcomes between follow-up periods, we completed subgroup analyses comparing responses from participants who had answered the same question in the two periods under comparison. We compared outcomes from the baseline (intake) survey—completed at the time of enrollment in the outreach program—to outcomes from follow-up surveys—completed after enrollment in the outreach program. Finally, to compensate for missing data, we conducted sensitivity analyses using worst case scenarios (where we replaced missing data with a negative outcome of no fixed housing, not being on OAT, not being engaged in care, and not abstinent from illicit opioids) for binary responses for each time period among those with any survey responses. Because the study was not designed to test specific hypotheses or find differences between groups, we did not formally calculate sample size.

## Results

By March 2020, when COVID restrictions precluded further in-person research, we had enrolled 84 participants, with a median age of 33, 29% identifying as female, and 32% reporting fixed housing. Overall, 31 (37%), 32 (38%), 33 (39%) and 34 (40%) completed the one-, two-, six-, and twelve-month follow-up questionnaires respectively (Table 1). 60 participants (71%) completed at least one follow-up questionnaire and were included in the follow-up analyses, eight of whom completed all four follow-up surveys. Of the 24 patients not completing follow-up questionnaires, ten had phone or in-person contact with the outreach team, two died, two were incarcerated during the entire follow-up period, two moved out of catchment area, and eight could not otherwise be contacted. Participants completing at least one follow-up questionnaire had a median of four encounters with the outreach team in the one-year follow-up period. Participants not completing follow-up questionnaires had similar baseline characteristics to those completing questionnaires, except for lower rates of housing and injection drug use.

**Table 1:** Participant baseline characteristics & follow-up encounters (*N* = 84)

Demographic characteristic	Whole sample (*N* = 84 unless stated otherwise)	Respondents to at least 1 survey (*N* = 60 unless stated otherwise)	Non-respondents (*N* = 24 unless stated otherwise)
Median age (years)	33 [IQR 28—40.3]	32.5 [IQR 29.5- 41]	32 [IQR 25.8 – 38]
Female (%) (1 respondent identified as other, the remainder as male)	24/84 (29%)	18/60 (30%)	6/24 (25%)
Identify as Indigenous or part Indigenous (%)	28/82 (34%)	19/58 (33%)	9/24 (38%)
Injection opioid use (%)	56/84 (67%)	42/60 (70%)	14/24 (58%)
Median baseline treatment motivation score (7-point scale)	5 [IQR 3.5–7] (*N* = 79)	5 [IQR 4–6.5] (*N* = 55)	5 [IQR 3–7]
Median Euroqol score	50 [IQR 30–70] (*N* = 83)	50 [IQR 30–70] (*N* = 59)	47.5 [IQR 30–70]
Fixed housing (%)	27/84 (32%)	24/60 (40%)	3/24 (12%)
Employed (%)	5/84 (6%)	4/60 (7%)	1/24 (4%)
Baseline engagement in care (%)	18/81 (22%)	13/58 (22%)	5/23 (22%)
Median anticipated helpfulness of outreach at baseline (7-point scale)	6 [IQR 5–7] (*N* = 83)	6 [IQR 5–7] (*N* = 59)	6 [IQR 5–7]
Median outreach team encounters in 1 year	3 [IQR 1–5.25] (Range 0–17)	4 [IQR 2–7] (Range 1–17)	0 [IQR 0–1] (Range 0–10)
Any outreach encounter	70/84 (83%)	60/60 (100%)	10/24 (42%)

No participants were on OAT at enrolment (by protocol), and the proportion of respondents on OAT changed to 42% at the one-month follow-up, 38% at two months, 48% at six months, and 56% at one year (Table 2). In the subgroup analysis, OAT increased between all encounters, except for the interval between the one-month and two-month follow-up (Table 3).

**Table 2:** Indicators at baseline and during follow up periods, including worst case-scenarios

	Intake survey	Intake survey among group with follow-up	One-month	One-month worst-case^a^	One-month worst-case^b^	Two- month	Two-month worst-case^a^	Two-month worst-case^b^	Six-month	Six-month worst -case^a^	Six-month worst -case^b^	Twelve-month	Twelve-month worst-case^a^	Twelve-month worst-case^b^
Number of respondents on OAT (%)	0/84 (0%)	0/60 (0%)	12/31 (39%)	12/60 (20%)	12/84 (14%)	12/32 (38%)	12/60 (20%)	12/84 (14%)	16/33 (48%)	16/60 (27%)	16/84 (19%)	19/34 (56%)	19/60 (32%)	19/84 (23%)
Number of respondents engaged in care (%)	18/81 (22%)	13/58 (22%)	20/31 (66%)	20/58 (34%)	20/81 (25%)	26/32 (81%)	26/58 (45%)	26/81 (32%)	24/33 (73%)	24/58 (41%)	24/81 (30%)	25/34 (74%)	25/58 (43%)	25/81 (31%)
Number of respondents with fixed housing (%)	27/84 (32%)	24/60 (40%)	16/31 (52%)	16/60 (27%)	16/84 (19%)	18/32 (56%)	18/60 (30%)	18/84 (21%)	16/33 (48%)	16/60 (27%)	16/84 (19%)	20/34 (59%)	20/60 (33%)	20/84 (24%)
Report no illicit opioid use in last 7 days (%)	0/81 (0%)	0/58 (0%)	4/31 (13%)	4/58 (7%)	4/81 (5%)	3/32 (9%)	3/58 (5%)	3/81 (4%)	5/33 (15%)	5/58 (9%)	5/81 (6%)	5/34 (15%)	5/58 (9%)	5/81 (6%)
Median days of illicit opioid use in last 7 days	7 [IQR 7–7] (*N* = 81)	7 [IQR 7–7] (*N* = 58)	7 [IQR 5.5–7] (*N* = 31)	N/A	N/A	7 [IQR 6.75–7] (*N* = 32)	N/A	N/A	7 [IQR 2–7] (*N* = 33)	N/A	N/A	7 [IQR 4.25–7] (*N* = 34)	N/A	N/A
Median Euroqol score	50 [IQR 30–70] (*N* = 83)	50 [IQR 30–70] (*N* = 59)	60 [IQR 40–65] (*N* = 30)	N/A	N/A	50 [IQR 40–70] (*N* = 32)	N/A	N/A	62.5 [IQR 50–75] (*N* = 32)	N/A	N/A	60 [IQR 45–75] (*N* = 33)	N/A	N/A

^a^Worst-case scenario if all participants completing at least 1 follow-up survey were not on OAT, not engaged in care, not housed, and not abstinent from illicit opioids

^b^Worst-case scenario if all participants completing an intake survey were not on OAT, not engaged in care, not housed, and not abstinent from illicit opioids

**Table 3:** Changes in indicators of interest from period to period: subgroup analysis including only participants responding in both periods under comparison

	*	0 to 1 month	0 to 2 months	0 to 6 months	0 to 12 months	1 to 2 months	1 to 6 months	1 to 12 months	2 to 6 months	2 to 12 months	6 to 12 months
Change in absolute % of respondents on OAT	Δ N1 N2	** + 39% ** 0/31 12/31	** + 38%** 0/32 12/32	** + 48%** 0 /33 16/33	** + 56%** 0/34 19/34	**-15%** 8/20 5/20	** + 5%** 8/19 9/19	** + 29%** 6/14 10/14	** + 5%** 9/19 10/19	** + 6%** 8/16 9/16	** + 17%** 8/18 11/18
Change in absolute % of respondents engaged in care	Δ N1 N2	** + 39%** 8/31 20/31	** + 70%** 5/30 26/30	** + 44%** 9/32 23/32	** + 58%** 5/33 24/33	** + 10%** 14/20 16/20	** + 5%** 12/19 13/19	** + 7%** 8/14 9/14	**-11%** 14/19 12/19	**-13%** 12/16 10/16	** + 6%** 13/18 14/18
Change in absolute % of respondents with fixed housing	Δ N1 N2	** + 10%** 13/31 16/31	** + 13%** 14/30 18/30	** + 10%** 13/30 16/30	** + 29%** 10/34 20/34	**0%** 14/20 14/20	+5% 10/19 11/19	** + 29%** 6/14 10/14	+16% 9/19 12/19	+19% 8/16 11/16	+22% 8/18 12/18
Change in absolute % no illicit opioid use for last 7 days	Δ N1 N2	** + 10%** 0/29 3/29	** + 9%** 0/32 3/32	** + 16%** 0/32 5/32	** + 13%** 0/32 4/32	**-5%** 1/20 0/20	** + 0%** 3/19 3/19	** + 7%** 1/14 2/14	**-11%** 3/19 1/19	**0%** 2/16 2/16	**+6%** 2/18 3/18
Median change in individual days of illicit opioid use in last 7 days	Δ IQ IQ M1 M2 N	**0** [0 – 0] 7 7 (*N* = 29)	**0** [0 – 0 7 7 (*N* = 32)	**0** [-1 – 0] 7 7 (*N* = 32)	**0** [-2.3 – 0] 7 7 (*N* = 32)	**0** [0 – 0 7 7 (*N* = 20)	**0** [0 – 0 7 7 (*N* = 19)	**0** [0 – 0 7 7 (*N* = 14)	**0** [0 – 0 7 7 (*N* = 19)	**0** [0 – 0 7 7 (*N* = 16)	**0** [0 – 0 7 7 (*N* = 18)
Median change in individual Euroqol quality of life score	Δ IQ IQ M1 M2 N	** + 1** [-11.5 – 16.5] 50 60 (*N* = 30)	** + 5** [ -10 – 20] 50 50 (*N* = 32)	** + 12.5** [0 – 30] 42.5 62.5 (*N* = 32)	** + 10** [0 – 30] 50 60 (*N* = 33)	** + 10** [-5– 15] 48.5 50 (*N* = 20)	** + 7.5** [-6.5 – 17.8] 60 67.5 (*N* = 18)	** + 5** [0 – 10] 60 65 (*N* = 13)	** + 10** [-2.5 – 19] 60 70 (*N* = 19)	**0** [-6.3 – 8.5] 60 65 (*N* = 16)	**-5** [-15 – 5] 70 65 (*N* = 17)

^*^Δ change, *N1* count at start of period, *N2* count at end of period, *IQ* interquartile range of change, *M1* median at start of period, *M2* median at end of period, *N* total respondents

Fifty eight percent of respondents were on OAT at some point in follow-up. Of the 62 separate instances of participants being on OAT at any time point, 58% were on methadone, 27% were on buprenorphine, and 10% were on sustained release oral morphine (Kadian) (Table 4).

**Table 4:** Types of OAT reported by participants at any time point

	Methadone	Buprenorphine	Sustained release oral morphine (Kadian)	Other
Number of respondents on this type of OAT (%)	36/62 (58%)	17/62 (27%)	6/62 (10%)	3/62 (5%) (1 on extended release morphine (M-Eslon), 1 on depot buprenorphine (Sublocade), 1 on injectable OAT)

At baseline, 22% of participants were engaged in addictions care, and this increased to 65% at one month, 81% at two months, 73% at six months, and 74% at one year (Table 2), with engagement consisting primarily of clinic visits. In the subgroup analysis, engagement in care increased from baseline to follow-up at all time periods, though it decreased between the two-month follow-up (the period with the highest engagement in care) and subsequent periods (Table 3).

The initial proportion of all participants with fixed housing was 32%, and 40% among participants with any follow-up. At follow-up, 48% to 59% of respondents had housing (Table 2). In the subgroup analysis, housing increased from baseline to follow-up at all time periods, and stayed stable or increased between each successive time period (Table 3). Only four respondents initially housed at baseline lost housing at some point during the study period, while 15 gained housing.

The median baseline Euroqol score was 50 and ranged from 50 to 60 at follow-up (Table 2). In the subgroup analysis, individual Euroqol quality of life scores rose more often than not in all periods compared to baseline, only dropping more often than not in the last six-month interval examined (Table 3).

At baseline and during all follow-up surveys, participants reported a median of seven days of illicit opioid use in the last week (Table 2). In the subgroup analysis, there was no change in median days of use overall, or in individuals reporting a change in use between periods (Table 3). No participants reported illicit opioid abstinence for a full week at baseline. At follow-up, 9% to 15% of respondents reported abstinence for the last seven days. In the subgroup analysis, changes in abstinence ranged from a decrease of 5% to an increase in 16% during the follow-up period.

The median score for perceived helpfulness of the outreach program was 6 out of 7 (very helpful) at baseline (Table 2), and 5 out of 7 (somewhat helpful, IQR 4–6) at the final survey encounter among respondents (*N* = 60).

**Fig. 1:**
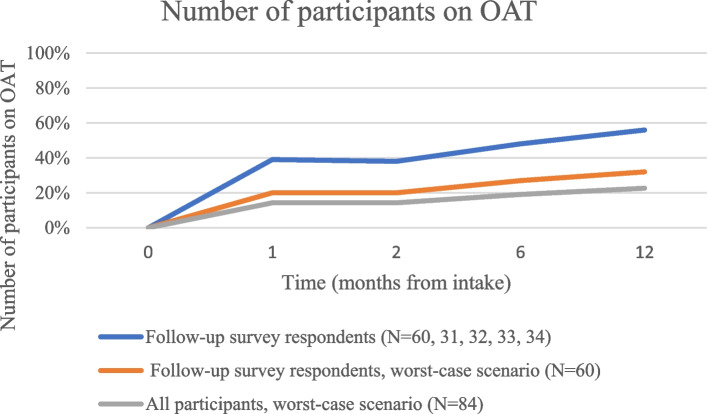
Number of participants on OAT

**Fig. 2:**
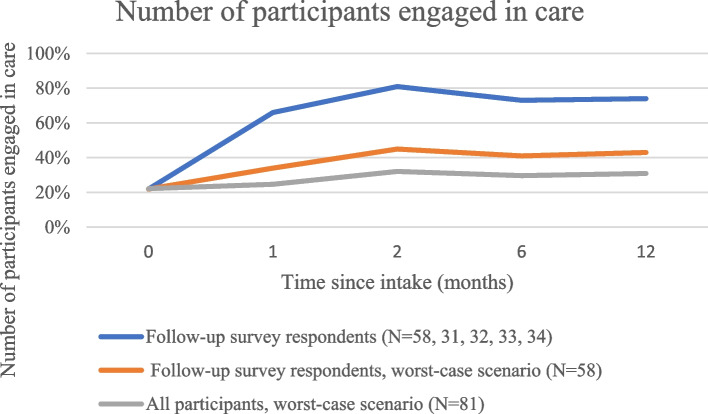
Number of participants engaged in care

**Fig. 3:**
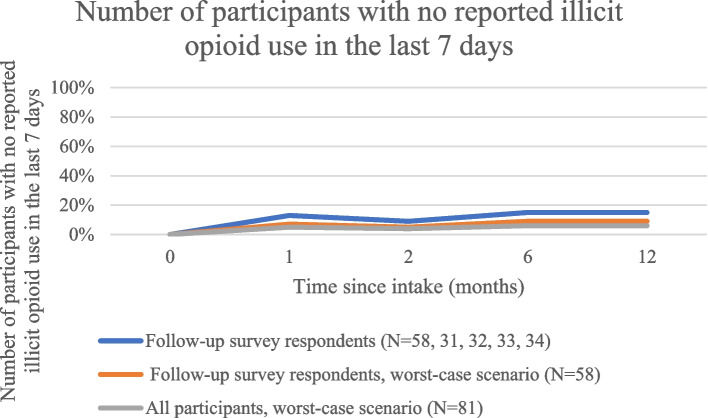
Number of participants with no reported illicit opioid use in the last 7 days

**Fig. 4:**
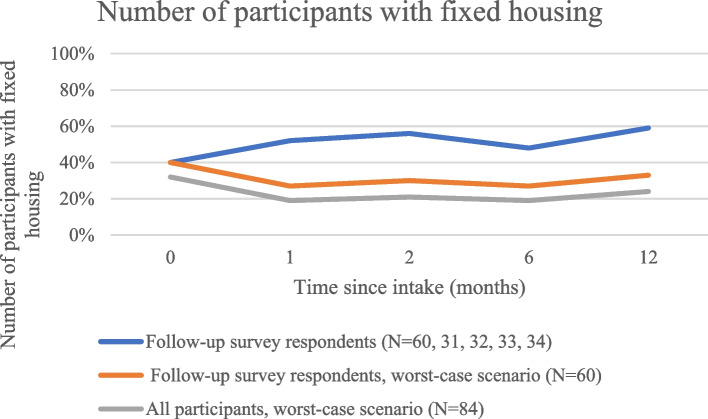
Number of participants with fixed housing

In the worst-case scenario analysis, the proportion on OAT (Fig. 1), proportion engaged in care (Fig. 2), and proportion abstinent from opioids in the last seven days (Fig. 3) still rose above baseline levels for all time periods. The proportion of those with fixed housing, however, decreased in the worst-case scenario (Fig. 4, Table 2).

## Discussion

This novel ED-initiated community outreach program for patients with opioid use disorder was associated with an increase in self-reported opioid agonist therapy uptake, engagement in addictions care, and housing at all time periods during the one-year follow-up. At each follow-up, participants reported that their quality of life and days of illicit opioid use remained stable. Overall, respondents found the program to be at least somewhat helpful at all times.

Given the chronic and relapsing nature of substance use disorders, ED interventions coupled with longer-term outreach programs will likely be more successful than ED interventions alone in improving the care of people with OUD. Our study demonstrates that continuing outreach for one year is associated with sustained increases in OAT uptake, ongoing engagement in addictions care, and stable housing. Our results thus differ from prior research with shorter-term interventions and follow-up. A randomized trial comparing only a pamphlet; a pamphlet with a referral to addictions treatment; and a pamphlet with a referral and two follow-up calls did not provide sustained benefit at one year [14]. Another randomized trial compared an intensive primary care follow-up program for patients initiated on buprenorphine in the ED to a referral or a brief intervention. The ED-initiated buprenorphine group had higher rates of addictions treatment and fewer days of illicit opioid use at two months compared to those not receiving ED buprenorphine [7]. However, this benefit was not observed at six and twelve months once intensive follow-up was discontinued [7]. Finally, patients with OUD who were randomized to a maximum of six sessions of case management did not have an increase in OAT initiation or engagement at three months [15]. Encouragingly, an evaluation of an ED-initiated buprenorphine program with referral to an addictions clinic and ongoing follow-up for six months demonstrated 35% patient adherence to treatment at six months, although its small size and retrospective nature limit generalizability; in addition, longer-term benefits are unclear [16]. One study found that collaborative care, including a “warm handoff” and follow up from a care coordinator, increased the likelihood that patients would start behavioural treatment for substance use disorders, and that at least one behavioural treatment session increased the likelihood of receiving pharmacological addictions treatment [17]. The same study noted that receiving pharmacological treatment (extended-release injectable naltrexone for alcohol use disorder or buprenorphine/naloxone for OUD) increased the likelihood of receiving the behavioural treatment [17]. A randomized controlled trial compared a “multi-component assertive intervention” to standard therapy in young adults with opioid use disorder who had been started on extended-release naltrexone. Standard therapy involved an outpatient addictions care referral. The intervention included: home delivery of extended-release naltrexone; family engagement through in-person, telephone, or text message coaching; contingency management; and treatment reminders and check-ins. The multi-component intervention was associated with more treatment doses, lower rates of relapse, and longer times to relapse compared to standard treatment [18]. Our prospective assessments extends these findings by suggesting benefits up to one year across multiple domains.

The variable effects of outreach interventions suggests that the duration, type, and association with OAT initiation may all influence their effectiveness for people with OUD. The OAT uptake noted in our study may reflect the expertise of the overdose outreach team involved, its ability to connect with patients in person, and the relative abundance of community addictions-related resources.

Despite consistently positive results across several study metrics, participants reported unchanged quality of life. It is therefore unclear whether participants—many of whom likely experienced food and shelter insecurity among other social and medical challenges—truly had a stable quality of life, or whether this could relate to the day-to-day variability and subjectivity of the Euroqol score. Alternatively, the Euroqol score might not have been the most appropriate tool to measure health-related quality of life in our study population.

This analysis of self-reported outcomes is one component of a larger study underway, in which pharmacy and health records of ED patients receiving outreach and other ED interventions will be reviewed over time and compared to those of ED patients not receiving those interventions. Objective data from chart reviews and provincial databases, as well as comparison with a control group, may corroborate self-reported outcomes described here. More research into outreach interventions of varying intensity and duration across multiple settings will stand the best chance of informing the implementation of outreach programs that will most benefit ED patients with OUD.

## Limitations

Our findings in an urban environment well-resourced with addictions services may not be generalizable to all settings. We did not achieve a high response rate at any set encounter, although nearly three quarters of participants provided at least one response. Non-responders may have differed from responders: they were less often housed, and unmeasured variables may have also factored. Non-responders may also have had worse outcomes over one year, which would increase the risk of positive bias in our results. Though different participants responded at different times, our subgroup analysis mitigates concerns about lost continuity, and our sensitivity analysis of a worst-case scenario still demonstrates improvement across most metrics.

As with any survey, recall and desirability biases may affect results, although self-reported substance use has been reliable in other settings [19]. In addition, self-reported OAT uptake may not reflect the more desired outcome of OAT retention. It is also possible that the evaluation of housing status, OAT status, and engagement in care at discrete time points may not accurately evaluate a participant’s overall situation over an entire year. Finally, study outcomes may themselves have influenced the completion of follow-up surveys; to illustrate, patients engaged in treatment, on OAT, and housed may have been more accessible for follow-up. That said, all participants responding to surveys received the outreach intervention and can therefore be viewed as providing the basis for a per-protocol analysis. Importantly, among those receiving outreach services, OAT uptake, engagement in care, and housing were all improved over baseline.

## Conclusion

An ED-initiated intensive one-year outreach program is associated with more OAT uptake, higher engagement in care, and stabler housing among people with OUD not initially on OAT. While the results from this study are promising, validation in diverse settings would be valuable. Intensive outreach programs and other ED efforts to improve transitions of care will likely benefit patients with OUD. Given the promise of health and social benefits, this study suggests that EDs can and should incorporate outreach into their care pathways for people with OUD.

### Supplementary Information

**Additional file 1.** Intake and follow-up questionnaire for intensive overdose outreach team follow-up program. 

## Data Availability

The datasets created and analyzed are available from the corresponding author upon reasonable request. The data is not publicly available because original consent from participants did not include consent to release data publicly beyond the study team.

## Abbreviations

OUD: Opioid use disorder
ED: Emergency department
OAT: Opioid agonist therapy
BC: British Columbia
RODS: Rapid Opioids Dependence Screen
SRO: Single-room occupancy
