# Supplementary material for: An emergency-department-initiated outreach program for patients with opioid use disorder is associated with an increase in agonist therapy and engagement in addictions care: a one-year cohort study
Source: Subst Abuse Treat Prev Policy. 2024 Feb 21;19:14. doi: 10.1186/s13011-023-00578-3 (PMC10880351; doi:10.1186/s13011-023-00578-3)
Supplement: Supplementary file 1 — Additional file 1. Intake and follow-up questionnaire for intensive overdose outreach team follow-up program. [file 13011_2023_578_MOESM1_ESM.pdf]

Research personnel (RP) to administer the following survey to participants. RP complete section A-I for all non-admitted ED patients approached (regardless of survey participation)

Primary Screening Section *(to be completed before and after encounter, not during)*

A. *Criteria for approaching potential participant. (Check all that apply)*
Initial Presentation (from tracking board, triage note, ambulance note)

1☐ Suspected or known opioid overdose (e.g., awoke with naloxone)
2☐ Suspected or known opioid withdrawal
3☐ Requesting opioid detoxification or rehabilitation
The following presentations also require one of the opioid use criteria below
4☐ Soft tissue infection consistent with IDU complication (abscess, cellulitis)
5☐ Exacerbation of chronic pain (including requesting analgesic refill)
6☐ Other reason related to opioid use. Please specify:________________________
7☐ Mental health presentation
8☐ Substance use/intoxication presentation not clearly related to opioids
9☐ Medical chief complaint not directly related to opioid use. Please specify:______________
10☐ blank

B. *Opioid Use Criteria: check all that apply; screen chart documents including standard ED medication list (Pharmanet print-out), triage note, ambulance note.*

1☐ On methadone or buprenorphine/naloxone from Pharmanet review within last 12 months
2☐ On prescribed opioid analgesics (in Pharmanet) at >100 mg morphine equivalent/day (67 mg oxycodone, 20 mg hydromorphone, 667 mg codeine, ≥ 25 mcg/hr fentanyl patch) within last 6 months
3☐ History of IDU (self-reported in triage note or ambulance note)
4☐ Self-disclosed use of any oral or smoked opioids in triage note or ambulance note
5☐ No evidence opioid use in screening documents beside presentations A1,A2, A3 above.
6☐ Referral to study team from ED staff member 7☐ Blank

C. Age: ______ ***C1. Assigned Study ID No:***___________________ ***(if agreed to participate)***

D. Sex: 1☐ Female 2☐ Male 3☐Other:____________________________

E. Face sheet address : 1☐ VCH 2☐ non-VCH 3☐ Unknown (clarify if interested in study)

F. Exclusion: *See criteria for opioid use disorder (OUD) and opioid withdrawal on page 2*
1☐Already took survey 2☐ Too sedated/confused/agitated/aggressive/psychotic/medically ill
3☐ in opioid withdrawal 4☐ Does not meet criteria for OUD 5☐ Later admitted to hospital
6☐ Pregnant 7☐ BTG not option per ED team (contraindications/other reasons)
8☐ Lives outside VCH catchment area 9☐ Currently on OAT (within last 5 days)
10☐ non-English speaking 11☐ No appropriate space to interview patient during visit

H. Declined to participate in survey
1☐ Refused 2☐ Withdrew during/after intake questions 3☐ Identified as study candidate, left before approached

I. Outcome (whether or not participated in survey)
1☐ Accepted BTG and agreed to iOOT participation 2☐ Outcome 1, but left without BTG
3☐ Declined BTG and agreed to iOOT participation 4☐ Outcome 5, but left without BTG
5☐ Accepted BTG and declined iOOT participation *(record refusal, remove all personal identifiers)*
6☐ Declined BTG and declined iOOT participation *(record refusal, remove all personal identifiers)*
7☐ Other, please specify8☐excluded9☐Accepted, but alternate plan w/ AAN (RAAC/other) or jailed

J. ED direct to RAAC? 1☐ yes 2☐ no(daytime) 3☐ no(RAAC closed) 4☐ unknown 5☐ n/a VGH

In general instructions for research personnel are in bold or italics (not to be read out loud)

1. ***Entry Questions*** *(The first question may be asked in a public location.)*
   a. "Have you taken this survey before?" *(i.e., prior participation)*
   1☐ Yes 2☐ No *(if "yes", stop here, and thank patient for their time)*

<u>FOLLOWING QUESTIONS FOR PRIVATE LOCATION OUT OF EAR SHOT OF OTHER PATIENTS.</u>

b. "Before we go through the full questionnaire, we would like to confirm you are eligible for the program. Within the last 5 days, have you been on treatment for opioid use disorder (with methadone, methadose Suboxone, or monitored injectable opioids)?"
1☐ Yes 2☐ No *(if "yes", stop here, and thank patient for their time)*

c. "What part of the Vancouver area do you live in?" *(VCH residence necessary for eligibility)*
1☐ Downtown East-Side 2☐ Other Vancouver area 3☐ North Vancouver 4☐ Richmond
5☐ West Vancouver 5☐ Other *(if "other", stop here, and thank patient for their time)*

Following questions adapted from RODS (Wickersham 2015)

d. "Have you used opioids within the last month?"
(Opioids include prescription pain medications such as oxycodone (oxycontin), codeine, hydrocodone (norco/vicodin), morphine, hydromorphone (dilaudid), medications used to treat addiction, such as methadone and suboxone, and street drugs such as heroin and fentanyl.
1☐ yes 2☐ no *(if no, stop here, and thank patient for their time, if yes, score 1 point)*

e. "Do you ever need to use more opioids to get high as when you first started using opioids?"
1☐ yes 2☐ no *(if yes, score 1 point)*

f. "Does the idea of missing a fix (or dose) ever make you anxious or worried?"
1☐ yes 2☐ no *(if yes, score 1 point)*

g. "In the morning, do you ever use opioids to keep from feeling "dopesick" or do you ever feel "dopesick"?"
1☐ yes 2☐ no *(if yes, score 1 point)*

h. "Do you worry about your use of opioids?"
1☐ yes 2☐ no *(if yes, score 1 point)*

i. "Do you find it difficult to stop or to not use opioids?"
1☐ yes 2☐ no *(if yes, score 1 point)*

j. "Do you ever need to spend a lot of time/energy on finding opioids or recovering from feeling high?"
1☐ yes 2☐ no *(if yes, score 1 point)*

k. "Do you ever miss important things like doctor's appointments, activities with family/friends, or other things because of opioids?"
1☐ yes 2☐ no *(if yes, score 1 point)*

If respondent scores <4 points in section c-j, the patient may not have OUD. STOP HERE.

l. "Do you feel very "dopesick" right now?"
1☐ yes 2☐ no 3☐ unsure **If yes, ask** "Are you interested in starting Suboxone right now?"

If patient states yes, and interested in starting Suboxone in ED, STOP HERE, & notify ED nurse, Addiction Nurse, and/or ED physician. (Patient is candidate for immediate start).

2. ***Demographics: "*** The following questions on demographics are voluntary. They ask about characteristics that may help researchers identify groups without enough access to care."

a. "Please identify the group below that best describes you from the following choices:"
1☐ Indigenous 2☐ Asian 3☐ African-American 4☐ White/Caucasian 5☐ Latin/Hispanic
6☐ other (specify):____________________ 7☐ mixed 8☐ prefer not to answer
9☐ mixed including Indigenous

b. "What is your current employment status?" *("job" may be full-time or part-time)*
1☐ steady job 2☐ odd jobs 3☐ unemployed 4☐ in school 5☐ prefer not to answer

c. "What best describes your current housing status?"
1☐ on the street 2☐ shelter 3☐ couch surfing/friends/family 4☐ SRO hotel
5☐ your own house/apartment 6☐ other 7☐ prefer not to answer

d. "Have you been to in jail over the last year? If yes, how many times?"
1☐1 2☐ 2-5 4☐ more than 5 5☐ none 6☐ prefer not to answer

e. *If yes to question 2d.*: "What is the longest period you've spent in jail over the last year?"
1☐ <1 day 2☐ 1-7 days 3☐ > 7 days, < 1month 4☐ 1 month or more
5☐ prefer not to answer 6☐ not applicable

3. ***Current health and social status***

a. "Do you currently have a regular doctor or clinic that could start treatment for opioid use disorder if you were interested?"
1☐ no 2☐ yes 3☐ prefer not to answer

b. "Within the last year, how many times have you overdosed on opioids (bad enough to have to go to the hospital or get naloxone (Narcan) from a bystander?"
1☐ none 2☐ once 3☐ 2-5 times 4☐ >5 times 4☐ not sure 5☐ prefer not to answer

c. "Beside today, how many times were you in an emergency department in the last year?"
1☐ none 2☐ once 3☐ 2-5 times 4☐ >5 times 5☐ not sure 6☐ prefer not to answer

d. "Within the last week have you had a clinic visit during which you and your care provider discussed treatment for opioid use disorder?"
1☐ no 2☐ yes 3☐ prefer not to answer

e. "Have you been to a detox facility within the last year?"
1☐ no 2☐ yes, once 3☐ yes, more than once 4☐ prefer not to answer

f. *If yes to 3e.:* "Have you gone to detox within the last week?"
1☐ no 2☐ yes, once 3☐ prefer not to answer 4☐ not applicable

g. "Have you been in a residential treatment (rehab) program within the last year?"
1☐ no 2☐ yes, once 3☐ yes, more than once 4☐ prefer not to answer

h. *If yes to 3g.,* "Have you gone to rehab within the last week?"
1☐ no 2☐ yes 3☐ prefer not to answer 4☐ not applicable

i. "On the following scale, please mark on the scale what you fell your health state is today:"

Best imaginable health state

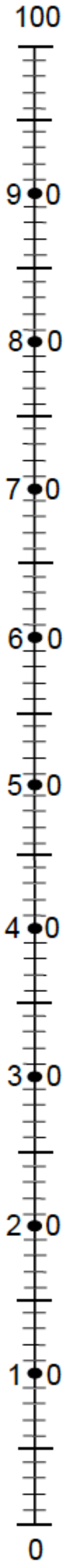

"To help people say how good or bad a health state is, we have drawn a scale (rather like a thermometer) on which the best state you can imagine is marked 100 and the worst state you can imagine is marked 0."

"We would like you to indicate on this scale how good or bad your own health is today, in your opinion. Please do this by drawing a line at whichever point on the scale indicates how good or bad your health is today."

Source: Adapted from EQ-5D-3L VAS

UK (English) © 1990 EuroQol Group EQ-5D™ is a trade mark of the EuroQol Group

Worst imaginable health state

4. ***Opioid and other drug/alcohol use:*** *"*The following questions ask about opioid use history, and the use of other substances such as alcohol and other drugs."
   a. "For how long have you used opioids?"
   $_1$☐ < 1 yr $_2$☐ 1 up to 5 yrs $_3$☐ 5 up to 10 yrs $_4$☐ ≥10 yrs $_5$☐ prefer not to answer

   b. "In the last year, what is the longest period of time you have gone without opioids (not counting time in jail, but counting time on treatment like methadone or Suboxone)"?
   $_1$☐ < 7 days $_3$☐ 1-4 wks $_4$☐ 1-3 mos $_5$☐ 3-6 mos $_5$☐ >6mos $_6$☐ prefer not to answer

   c. "When you use opioids, how do you use them?" *(check all that apply)*
   $_1$☐ inject $_2$☐ smoke/snort $_3$☐ swallow $_4$☐ prefer not to answer $_5$☐ other____________

   d. "What is the way you use opioids the most often?"
   $_1$☐ inject $_2$☐ smoke/snort $_3$☐ swallow $_4$☐ prefer not to answer $_5$☐ other____________

For questions e.-l.), use timeline follow back method as a cue, starting from today's day:
(use calendar or index card with days of week if needed to aid participant recall)

   e. "How many days ago did you last use opioids? (Or hours ago if you used in last 24 hours?)"
   $_1$☐ 1 $_2$☐ 2 $_3$☐ 3 $_4$☐ 4 $_5$☐ 5 $_6$☐ 6 $_7$☐ 7 $_8$☐ > 7 $_9$☐ ________hrs ago $_{10}$☐ prefer not to answer

   f. "In the last 7 days, how many days have you used opioids?"
   $_1$☐ 1 $_2$☐ 2 $_3$☐ 3 $_4$☐ 4 $_5$☐5 $_6$☐ 6 $_7$☐ 7 $_8$☐ none $_9$☐ unsure $_{10}$☐ prefer not to answer

   g. "In the last 7 days, how many days have you used amphetamines?"
   $_1$☐ 1 $_2$☐ 2 $_3$☐ 3 $_4$☐ 4 $_5$☐5 $_6$☐ 6 $_7$☐ 7 $_8$☐ none $_9$☐ unsure $_{10}$☐ prefer not to answer

   h. "In the last 7 days, how many days have you used crack or powdered cocaine?"
   $_1$☐ 1 $_2$☐ 2 $_3$☐ 3 $_4$☐ 4 $_5$☐5 $_6$☐ 6 $_7$☐ 7 $_8$☐ none $_9$☐ unsure $_{10}$☐ prefer not to answer

   i. "In the last 7 days, how many days have you used alcohol?"
   $_1$☐ 1 $_2$☐ 2 $_3$☐ 3 $_4$☐ 4 $_5$☐5 $_6$☐ 6 $_7$☐ 7 $_8$☐ none $_9$☐ unsure $_{10}$☐ prefer not to answer

   j. "In the last 7 days, how many days have you intentionally used benzos (Ativan/Valium)?"
   $_1$☐ 1 $_2$☐ 2 $_3$☐ 3 $_4$☐ 4 $_5$☐5 $_6$☐ 6 $_7$☐ 7 $_8$☐ none $_9$☐ unsure $_{10}$☐ prefer not to answer

   k. "In the last 7 days, how many days have you used marijuana?"
   $_1$☐ 1 $_2$☐ 2 $_3$☐ 3 $_4$☐ 4 $_5$☐5 $_6$☐ 6 $_7$☐ 7 $_8$☐ none $_9$☐ unsure $_{10}$☐ prefer not to answer

   l. "In the last 7 days, how many days have you used another drug? Which drug:_______________"
   $_1$☐ 1 $_2$☐ 2 $_3$☐ 3 $_4$☐ 4 $_5$☐5 $_6$☐ 6 $_7$☐ 7 $_8$☐ none $_9$☐ unsure $_{10}$☐ prefer not to answer

5. Experience with opioid use disorder treatment and overdose prevention

   a. "Have you ever taken medication for opioid use disorder prescribed by a doctor?"
   $_1$☐ no $_2$☐ Suboxone $_3$☐ methadone $_4$☐ Methadose $_5$☐ Kadian (long acting morphine)
   $_6$☐ injectables $_7$☐ Naltrexone $_8$☐ prefer not to answer *(check all that apply)*

   b. *If yes to a):* "How many different times have you tried to start medication in the last year?"
   $_1$☐ $_2$☐ 2 $_3$☐ 3 $_4$☐ 4 $_5$☐ 5 $_6$☐ >5 $_7$☐ none $_8$☐ prefer not to answer

   c. *If yes to a):* "When was the last time you tried to start such a medication?"
   $_1$☐ within last month $_2$☐ 1-6 months ago $_3$☐ 6-12 months ago $_4$☐ more than 1 year ago

d. "If you ever tried Suboxone, did you ever get precipitated withdrawal? That means rapidly worsening dopesickeness if you take it too early": 1☐ yes 2☐ no 3☐ prefer not to answer

e. *If yes to a):* "Please rate your satisfaction with each medication you have previously taken:" (***Use index card with scale from 1"extremely dissatisfied" to 7 "extremely satisfied")***

<u>Suboxone</u>
1☐ extremely dissatisfied 2☐ very dissatisfied 3☐ somewhat dissatisfied 4☐ neither satisfied nor dissatisfied 5☐ somewhat satisfied 6☐ very satisfied 7☐ extremely satisfied

<u>methadone (Metadol)</u>
1☐ extremely dissatisfied 2☐ very dissatisfied 3☐ somewhat dissatisfied 4☐ neither satisfied nor dissatisfied 5☐ somewhat satisfied 6☐ very satisfied 7☐ extremely satisfied

<u>Methadose</u>
1☐ extremely dissatisfied 2☐ very dissatisfied 3☐ somewhat dissatisfied 4☐ neither satisfied nor dissatisfied 5☐ somewhat satisfied 6☐ very satisfied 7☐ extremely satisfied

<u>Kadian</u>
1☐ extremely dissatisfied 2☐ very dissatisfied 3☐ somewhat dissatisfied 4☐ neither satisfied nor dissatisfied 5☐ somewhat satisfied 6☐ very satisfied 7☐ extremely satisfied

<u>other:</u>______________________________
1☐ extremely dissatisfied 2☐ very dissatisfied 3☐ somewhat dissatisfied 4☐ neither satisfied nor dissatisfied 5☐ somewhat satisfied 6☐ very satisfied 7☐ extremely satisfied

f. At this moment, on a scale from 1-7, how motivated are you to start treatment for opioid use disorder? (from 1, not motivated at all, to 7, extremely motivated)

1☐ 2☐ 3☐ 4☐ 5☐ 6☐ 7☐
8☐ prefer not to answer

g. "Have you ever owned a take home naloxone (Narcan) kit?"
1☐ yes, currently have 2☐ previously had 3☐ no, never 4☐ prefer not to answer

h. "Have you ever used a take home naloxone (Narcan) kit?"
1☐ yes, on myself 2☐ yes, on others 3☐ yes, to both 4☐ no 5☐ prefer not to answer

i. "When using drugs, do you use overdose prevention or supervised injection sites?"
1☐ never 2☐ sometimes 3☐ most times 4☐ every time 5☐ prefer not to answer

j. "When using drugs, how often do you go to drug checking sites first?"
1☐ never 2☐ sometimes 3☐ most times 4☐ every time 5☐ prefer not to answer

6. Attitudes on emergency departments, buprenorphine/naloxone, & outreach
"The following questions ask about your opinion of using the emergency department as place to receive buprenorphine/naloxone (Suboxone) to go-packs and other services."

a. "Do you think emergency departments should give out Suboxone To-Go starter packs?"
1☐ yes 2☐ no 3☐ unsure 4☐ prefer not to answer

b. "Do you feel comfortable enough in this emergency department to receive Suboxone To-Go or other treatments, education, and resources related to opioid use disorder?"
1☐ yes 2☐ no 3☐ unsure 4☐ prefer not to answer

c. "Do you feel stigma around drug use gets in the way of your care in the emergency department?"
1☐ yes 2☐ no 3☐ sometimes 4☐ not sure 5☐ prefer not to answer

d. "As part of the program you are enrolling in, a community outreach team will be contacting several times over the next year you to see how you are doing and to help connect you with whatever services you might need. How helpful do you think this outreach will be to you?"

extremely unhelpful — somewhat unhelpful — somewhat helpful — extremely helpful

1☐ 2☐ 3☐ 4☐ 5☐ 6☐ 7☐

very unhelpful — Neither helpful/unhelpful — very helpful

8☐ prefer not to answer

<u>"On the same scale from extremely unhelpful to extremely helpful, please rate these other addiction-related emergency department services in terms of how helpful they are to you."</u>

e. "Receiving take-home naloxone (Narcan) kits:"

1☐ 2☐ 3☐ 4☐ 5☐ 6☐ 7☐

8☐prefer not to answer

f. "Buprenorphine/naloxone [Suboxone] to go packs to start treatment out of the emergency:"

1☐ 2☐ 3☐ 4☐ 5☐ 6☐ 7☐

8☐prefer not to answer

g. "Starting Suboxone during the emergency department stay:"

1☐ 2☐ 3☐ 4☐ 5☐ 6☐ 7☐

8☐prefer not to answer

h. "Starting other treatments for opioid use disorder in emergency (methadone, Kadian, etc):"

1☐ 2☐ 3☐ 4☐ 5☐ 6☐ 7☐

8☐prefer not to answer

i. "Getting missed doses of current medication (like methadone, Suboxone, Kadian, etc):"

1☐ 2☐ 3☐ 4☐ 5☐ 6☐ 7☐

8☐prefer not to answer

j. "Speaking to an addiction nurse or addiction specialist doctor:"

1☐ 2☐ 3☐ 4☐ 5☐ 6☐ 7☐

8☐prefer not to answer

k. "Are there other addiction-related services you would like to see offered in the emergency-department?" 1☐ yes, specify:__________________ 2☐ no 3☐ unsure 4☐ prefer not to answer

l. "If you are not interested in a Suboxone to-go pack today, please tell us the main reason" (choose from the following reasons below or give us another reason)."

1☐ I am not interested in treatment
2☐ I have been on Suboxone before and I didn't like it
3☐ I would not be able to wait long enough (and to be dopesick enough) to start it
4☐ I am afraid of going into precipitated withdrawal (rapidly worsening dopesickness)
5☐ I would not want to give up the feeling of getting high
6☐ I am more interested in starting another kinds of treatment (methadone, Kadian, etc.)
7☐ Other:____

m. "If you are interested in a Suboxone to-go pack today, please tell us the main reason (choose from the following reasons or give us another reason). To be clear, none of the reasons you might mention would prevent you from getting a starter pack today."

1☐ I want to start treatment later today when I am in withdrawal (dopesick)
2☐ It would be good to have around in case I want to start treatment later
3☐ I could use it in a pinch if I am dopesick and don't have other opioids on me
4☐ I could give it to a friend in need or sell it
5☐ Other:____

OOT staff to administer the following survey to participants.
OOT staff to complete section A-L even if unable to locate

A. ***Study ID number***:____________________ **A1. Enrollment Site:** 1☐ SPH 2☐ VGH

B. ***Age:*** ________

C. Gender
1☐ female 2☐ male 3☐ other_________________

D. ***BTG received at index ED visit***
1☐ yes 2☐ no

E. OOT follow up visit:
1☐ 1 month 2☐ 2month ☐ 6 month 2☐ 12 month

F. Contact Date *Actual:*__________________________*Targeted:*________________________

G. Number of attempts to contact
1☐ 1 2☐ 2 3☐ 3 4☐ 4

H. Successful Contact?
1☐ yes interval from last contact:_____________ (in months)
2☐ no minimum 3 tries over 2 weeks from target date required

I. Contact information change since last encounter?
1☐ yes 2☐ no ***(if yes, please update master study contact sheet)***

J. Additional OOT re-referrals since initial program enrollment
1☐ none 2☐ 1 3☐ 2 4☐ 3 5☐ more than 3 8☐ other:___________________

K. OOT Services provided
1☐ __________________________
2☐ __________________________
3☐ __________________________

L. Outcome
1☐ patient not found
2☐ patient completed questionnaire, compensation given
3☐ patient found but unable to complete questionnaire
4☐ patient expresses wish to withdraw from study
8☐ other, please specify___________________________

Sample script for OOT personnel administering the survey
"Hi. I am working with the overdose outreach team. On an emergency department visit, you enrolled in a study involving increased outreach from our team. As part of this study, we will be asking you some questions about your experiences. This should take about 10-15 minutes of your time. Because we recognize your time is valuable and because we value your input, we will pay you $20 at the completion of the questionnaire. Are you still willing to be part of this study? Participation in this study is completely voluntary: At any time, you could say 'I prefer not to answer this question.' Thank you for your help!"

Instructions to research staff (not to be read to participant aloud) in bold or italics below:

1. ***BTG: Buprenorphine/naloxone [Suboxone] To-Go Starter Pack (Enter "not applicable" for 1a-1h if question already answered and if BTG no longer in possession at last OOT encounter)***

   a. "Did you receive a Suboxone starter pack when you first enrolled in this program in the emergency department?" ***(confirming recorded data above) (micro-dose in bubble pack)***
   1☐ yes, regular dose 2☐ yes, micro-dose 3☐ yes-unsure 4☐ no 5☐ not applicable

   b. "What did you do with your Suboxone starter pack?"
   1☐ used it 2☐ sold it 3☐ kept it for later 4☐ gave it a way 5☐ lost it
   6☐ other:____________________ 7☐ prefer not to answer 8☐ not applicable

   c. "If you used your Suboxone starter pack, did you get precipitated withdrawal (that's when dope sickness becomes much worse quickly because you used Suboxone too early)?"
   1☐ yes 2☐ no 3☐ prefer not to answer 4☐ not applicable

   d. *If yes to c*: "How did you deal with the situation (precipitated withdrawal)?"
   1☐ stopped Suboxone, waited it out 2☐ stopped Suboxone, self-treated with other opioid
   3☐ continued Suboxone on my own 4☐ sought medical care, then continued Suboxone
   5☐ sought medical care, then stopped 6☐ prefer not to answer 7☐ not applicable

   e. "If you used your Suboxone starter pack, did you then go get more Suboxone doses?"
   1☐ yes, clinic (specify):__________________ 2☐ yes, hospital/ED (specify):____________________
   3☐ no 4☐ prefer not to answer 5☐ not applicable

   f. *If yes to e)* "Were you able to stay on Suboxone without a major break until today? (major break means where you missed several days in a row and had to start again from scratch)"
   1☐ yes 2☐ no 3☐ prefer not to answer 4☐ not applicable

   g. "If you were unable to start/stay on Suboxone, choose the reason that best explains why:"
   1☐ still felt dopesick 2☐ still had too much craving 3☐ did not like the way I felt
   4☐ I had trouble finding a clinic to continue it 5☐ daily dispense not convenient
   5☐ not really ready for treatment 7☐ starting instructions too complicated
   8☐ prefer not to answer 9☐ not applicable 10☐ other:__________________________

   h. "If you were able to stay on Suboxone, please choose the reason that best explains why"
   1☐ craving controlled 2☐ felt better overall 3☐ ready to stop opioids 4☐ court-ordered
   5☐ child custody issue 6☐ prefer not to answer 7☐ not applicable 8☐ other:_____________________

2. ***Opioid and other drug/alcohol use (Note: OAT or iOAT would not be "street opioids")***

   a. "Since the study team last contacted you, what is the longest period of time you have gone without street opioids?" "If some of that time was in jail, please state how much of it:"_______
   1☐ < 1 wk 3☐ 1-4 wks 4☐ 1-3 mos 5☐ 3-6 mos 5☐ >6mos 6☐ prefer not to answer

   b. "Since the study team last contacted you, have you stopped using street opioids completely*?*"
   1☐ no 2☐ <1 wk 3☐ 1-4 wks 4☐ 1-3 mos 4☐ 3-6 mos 5☐ >6mos 6☐ prefer not to answer

   For questions 2c-2i, use timeline follow back method as a cue, starting from today's day: (use calendar or index card with days of week if needed to aid participant recall)

   c. "When was the last time you used opioids? (Or hours ago if you used in last 24 hours?)"
   1☐ 1 2☐ 2 3☐ 3 4☐ 4 5☐ 5 6☐ 6 7☐ 7 8☐ > 7 9☐ _______hrs ago 10☐ prefer not to answer

   d. "In the last 7 days, how many days have you used opioids?"
   1☐ 1 2☐ 2 3☐ 3 4☐ 4 5☐5 6☐ 6 7☐ 7 8☐ none 9☐ unsure 10☐ prefer not to answer

e. "In the last 7 days, how many days have you used crystal meth or other amphetamines?"
$_1$☐ 1 $_2$☐ 2 $_3$☐ 3 $_4$☐ 4 $_5$☐5 $_6$☐ 6 $_7$☐ 7 $_8$☐ none $_9$☐ unsure $_{10}$☐ prefer not to answer

f. "In the last 7 days, how many days have you used cocaine (crack or powder)?"
$_1$☐ 1 $_2$☐ 2 $_3$☐ 3 $_4$☐ 4 $_5$☐5 $_6$☐ 6 $_7$☐ 7 $_8$☐ none $_9$☐ unsure $_{10}$☐ prefer not to answer

g. "In the last 7 days, how many days have you used alcohol?"
$_1$☐ 1 $_2$☐ 2 $_3$☐ 3 $_4$☐ 4 $_5$☐5 $_6$☐ 6 $_7$☐ 7 $_8$☐ none $_9$☐ unsure $_{10}$☐ prefer not to answer

h. "In the last 7 days, how many days have you intentionally used benzos (Ativan/Valium)?"
$_1$☐ 1 $_2$☐ 2 $_3$☐ 3 $_4$☐ 4 $_5$☐5 $_6$☐ 6 $_7$☐ 7 $_8$☐ none $_9$☐ unsure $_{10}$☐ prefer not to answer

i. "In the last 7 days, how many days have you used marijuana?"
$_1$☐ 1 $_2$☐ 2 $_3$☐ 3 $_4$☐ 4 $_5$☐5 $_6$☐ 6 $_7$☐ 7 $_8$☐ none $_9$☐ unsure $_{10}$☐ prefer not to answer

j. "In the last 7 days, how many days have you used another drug? Which drug:_______________"
$_1$☐ 1 $_2$☐ 2 $_3$☐ 3 $_4$☐ 4 $_5$☐5 $_6$☐ 6 $_7$☐ 7 $_8$☐ none $_9$☐ unsure $_{10}$☐ prefer not to answer

3. ***Current health and social status update***

a. "What is your current employment status? *("job" may be part-time or full-time)"*
$_1$☐ steady job $_2$☐ odd jobs $_3$☐ unemployed $_4$☐ in school $_4$☐ prefer not to answer

b. "What best describes your current housing status?"
$_1$☐ on the street $_2$☐ shelter $_3$☐ couch surfing/friends/family $_4$☐ SRO hotel
$_5$☐ your own house/apartment $_6$☐ other $_7$☐ prefer not to answer

c. "Have you been to jail since you were last contacted by the study team? How many times?"
$_1$☐1 $_2$☐ 2-5 $_4$☐ more than 5 $_5$☐ none $_6$☐ prefer not to answer

d. *If yes to question 3c.*: "What is the longest time you have spent in jail during this period?"
$_1$☐ <1 day $_2$☐ 1-7 days $_3$☐ > 7 days, < 1month $_4$☐ 1 month or more
$_5$☐ prefer not to answer $_6$☐ not applicable

e. "Since the study team last contacted you, have you overdosed on opioids (bad enough to have to go to the hospital or get naloxone (Narcan) from a bystander?"
$_1$☐ no $_2$☐ once $_3$☐ 2-5 times $_4$☐ >5 times $_4$☐ not sure $_5$☐ prefer not to answer

f. "Within the last week have you had a clinic visit during which you and your care provider discussed treatment for opioid use disorder?"
$_1$☐ yes $_2$☐ no $_3$☐ prefer not to answer

g. "Since the study team last contacted you, have you been to a detox facility?"
$_1$☐ no $_2$☐ yes, once $_3$☐ yes, more than once $_4$☐ prefer not to answer

h. *If yes to 3g.:* "Have you been to detox within the last week?"
$_1$☐ no $_2$☐ yes $_3$☐ prefer not to answer $_4$☐ not applicable

i. "Since the study team last contacted you, have you been in a residential treatment (rehab)?"
$_1$☐ no $_2$☐ yes, once $_3$☐ yes, more than once $_4$☐ prefer not to answer

j. *If yes to3i.*: "Have you been in rehab within the last week?"
$_1$☐ no $_2$☐ yes $_3$☐ prefer not to answer $_4$☐ not applicable

k. “On the following scale, please mark on the scale what you feel your health state is today:”

Best imaginable health state

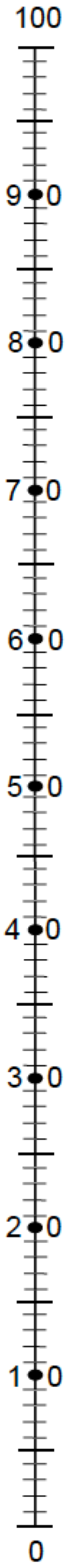

“To help people say how good or bad a health state is, we have drawn a scale (rather like a thermometer) on which the best state you can imagine is marked 100 and the worst state you can imagine is marked 0.”

“We would like you to indicate on this scale how good or bad your own health is today, in your opinion. Please do this by drawing a line at whichever point on the scale indicates how good or bad your health is today.”

Source: EQ-5D-3L VAS

UK (English) © 1990 EuroQol Group EQ-5D™ is a trade mark of the EuroQol Group

Worst imaginable health state

4. Experience with opioid use disorder treatment (update since last encounter)

a. "Are you currently on a medication for opioid use disorder that is prescribed by a doctor?"
1☐ no 2☐ Suboxone 3☐ methadone 4☐ Methdadose 5☐ Kadian (long acting morphine)
6☐ injectables 7☐ naltrexone 8☐ prefer not to answer 9☐ other_______________

b. *If yes to 4a.:* "How many days ago was your last dose of medication?"
1☐ 0 (today) 2☐ 1 3☐ 2 4☐ 3 5☐ 4 6☐ 5 7☐ 6 8☐ ≥7 8☐ prefer not to answer

c. *If yes to 4a.:* "How long have you now been on treatment?"
1☐ less than 1 week 2☐ 1 week up to a month 3☐ 1-3 months 4☐ 3-6 months 5☐ 6 months or more 6☐ not applicable 7☐ prefer not to answer

d. "Since the study team last contacted you, what is the longest you have stayed on meds?"
1☐ less than 1 week 2☐ 1 week up to a month 3☐ 1-3 months 4☐ 3-6 months 5☐ 6 months or more 6☐ not applicable 7☐ prefer not to answer

e. "Please rate your satisfaction with the medication (s) you a☐ are or b☐ were taking since the last time you were contacted by the study team". ***(Use index card with scale 1-7)***

<u>Suboxone</u>
1☐ extremely dissatisfied 2☐ very dissatisfied 3☐ somewhat dissatisfied 4☐ neither satisfied nor dissatisfied 5☐ somewhat satisfied 6☐ very satisfied 7☐ extremely satisfied

<u>methadone (Metadol)</u>
1☐ extremely dissatisfied 2☐ very dissatisfied 3☐ somewhat dissatisfied 4☐ neither satisfied nor dissatisfied 5☐ somewhat satisfied 6☐ very satisfied 7☐ extremely satisfied

<u>Methadose</u>
1☐ extremely dissatisfied 2☐ very dissatisfied 3☐ somewhat dissatisfied 4☐ neither satisfied nor dissatisfied 5☐ somewhat satisfied 6☐ very satisfied 7☐ extremely satisfied

<u>Kadian</u>
1☐ extremely dissatisfied 2☐ very dissatisfied 3☐ somewhat dissatisfied 4☐ neither satisfied nor dissatisfied 5☐ somewhat satisfied 6☐ very satisfied 7☐ extremely satisfied

<u>other:_______________________________</u>
1☐ extremely dissatisfied 2☐ very dissatisfied 3☐ somewhat dissatisfied 4☐ neither satisfied nor dissatisfied 5☐ somewhat satisfied 6☐ very satisfied 7☐ extremely satisfied

f. "Since the last time you were contacted by the study team, how many different attempts have you made to start a medication for treatment?"
1☐ 1 2☐ 2 3☐ 3 4☐ 4 5☐ 5 6☐ >5 7☐ none 8☐ prefer not to answer

g. "Right now, on a scale from 1-7, how motivated are you to a☐ start or b☐ continue treatment for opioid use disorder? (from 1, not motivated at all, to 7, extremely motivated)"

1☐ 2☐ 3☐ 4☐ 5☐ 6☐ 7☐
8☐prefer not to answer

5. Opinion on outreach team services

a. "So far, how helpful do you think this outreach program has been for you?"
   1☐ extremely unhelpful   2☐ very unhelpful   3☐ somewhat unhelpful
   4☐ neither helpful nor unhelpful   5☐ somewhat helpful   6☐ very helpful
   7☐ extremely helpful

b. Do you have any suggestions to improve this program?
   If yes, please specify____

6. For VGH participants: 1 month follow-up only. Skip for follow-up at 2,4,6, & 12 months.

a. "If you received a Suboxone pack and did not use it, what were your reasons for not using it?"
   1☐ I wasn't ready   2☐ potential for withdrawal symptoms   3☐ financial gain
   4☐ other:____________ 5☐ prefer not to answer   6☐ not applicable

b. "If you stopped and then restarted Suboxone, where did you go to restart Suboxone?"
   1☐ clinic (specify):____________   2☐ hospital during admission (specify) ____________
   3☐ family doctor   4☐ emergency room(specify)____________________
   5☐ from a friend or non-healthcare source   7☐ other:____________________

c. "Do you think emergency departments should keep offering Suboxone to their patients?"
   1☐ yes   2☐ no   3☐ not sure   4☐ prefer not to answer

d. "Would you be willing to try starting Suboxone from an emergency department in the future?"
   1☐ yes   2☐ no   3☐ not sure   4☐ prefer not to answer

e. "How do you think it would be easiest for you to access Suboxone (if you wanted it)?
   1☐ family doctor   2☐ addiction clinic   3☐ emergency department
   4☐ not sure   5☐ prefer not to answer   6☐ other
   If other, please specify____
